# Revealing Disorder, Sorption Locations and a Sorption-Induced Single Crystal–Single Crystal Transformation in a Rare-Earth *fcu*-Type Metal–Organic Framework

**DOI:** 10.1021/acs.inorgchem.4c04286

**Published:** 2024-11-04

**Authors:** A. R. Bonity J. Lutton-Gething, Fajar I. Pambudi, Ben F. Spencer, Daniel Lee, George F. S. Whitehead, Inigo J. Vitorica-Yrezabal, Martin P. Attfield

**Affiliations:** † Department of Chemistry, School of Natural Sciences, The University of Manchester, Oxford Road, Manchester M13 9PL, U.K.; ‡ Department of Materials and National Graphene Institute, The University of Manchester, Oxford Road, Manchester M13 9PL, U.K.; § Photon Science Institute, The University of Manchester, Oxford Road, Manchester M13 9PL, U.K.; ∥ Department of Chemical Engineering, School of Engineering, The University of Manchester, Oxford Road, Manchester M13 9PL, U.K.

## Abstract

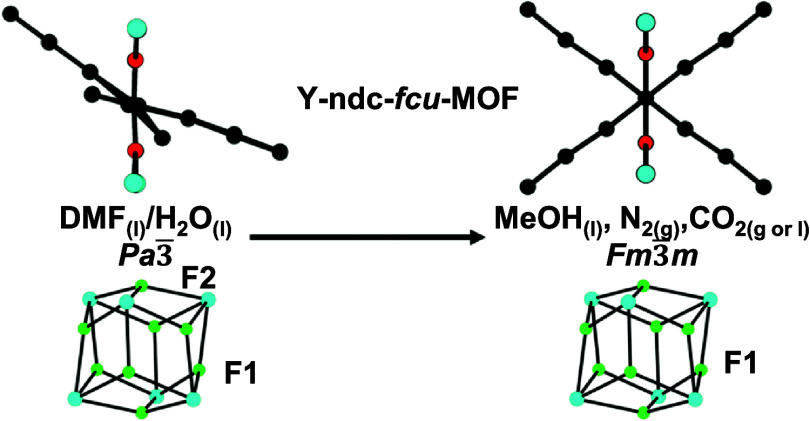

Rare-earth metal–organic frameworks (RE-MOFs) formed in the presence of fluoride donors are a group of complex and applicable MOFs. Determining structural complexity is crucial in applying such MOFs and has been achieved to uncover framework disorders in the important *fcu* framework topology MOF, Y-ndc-*fcu*-MOF (**1**). **1** is found to contain F^–^ groups disordered over the μ_3_-face-capping sites in its secondary building unit (SBU) and framework distortions upon sorption of different guest molecules. The favored location of the guests is within the octahedral cage of **1** where they interact with the Y^3+^ centers. The size, shape, and interactions of the different guests lead to subtle distortions within the SBU and adoption of specific orientations of the naphthalene group of the 1,4-naphthalenedicarboxylate framework linkers. The sorption of DMF_(l)_/H_2_O_(l)_ lowers the symmetry from cubic *Fm*3̅*m* (for MeOH_(l)_, N_2(g)_, CO_2(g or l)_) to cubic *Pa*3̅ (for DMF_(l)_/H_2_O_(l)_) symmetry with retention of the *fcu* topology, and conversion between the *Pa*3̅ and *Fm*3̅*m* structures is induced by solvent exchange. Such disorder and sorption locations and transformation are important considerations during the optimization and application of MOFs for sorption-based technologies.

## Abstract 2

Structural reinvestigation of the metal−organic framework Y-ndc-*fcu*-MOF finds μ_3_-F^−^ groups disordered over face-capping sites in its secondary building block (SBU) and framework distortions upon sorption of guest molecules. Guests are located within the octahedral framework cages, where they interact with the Y^3+^ centers. The different guests lead to subtle distortions within the SBU and specific orientations of the framework linker with an example of an accompanying *Fm*3̅*m* to *Pa*3̅ symmetry change of the *fcu* topology framework.

## Introduction

An exciting new group of functional metal–organic framework (MOF) that is emerging are rare-earth (RE) MOFs formed from synthesis mixtures containing modulators or linkers possessing terminal fluorine atoms.^1,2^ These RE-MOFs demonstrate great promise for numerous applications^3−10^ and possess complex framework structures that contain various degrees of disorder involving the constituent organic linker or the secondary building unit (SBU). Such disorder is particularly apparent in RE-MOFs containing the cuboctahedrally connected RE_6_X_a_R_12_ SBU that consists of six RE^3+^ ions organized in an octahedral or trigonal antiprismatic formation face-capped by μ_3_-X groups (X is potentially F^–^, (OH)^−^ or O^2–^, *a* ≤ 8) and edge-bridged by the bidentate groups of ditopic organic linkers, R.^11^ The three-dimensional framework is formed by the connection of the SBUs by the organic linkers. SBUs containing solely μ_3_-face-capping (OH)^−^ or F^–^ groups have been reported,^1,8^ in addition to those containing a mixture of μ_3_-face-capping (OH)^−^ and F^–^ groups,^12^ and those containing a combination of μ_3_-face-capping F^–^ groups and assumed (OH)^−^ or O^2–^ species.^13,14^ Disorder of the organic linker is observed through the adoption of different connection modes of the linker bidentate groups to the SBU^12,15^ and orientations of the noncoordinating spacer portion of the linker.^16^ The disorder involving the SBU can occur during initial synthesis of the MOF, while that involving the noncoordinating spacer of the linker will potentially depend upon any host–guest relationships within the void space of the framework. However, the latter has not been experimentally determined within this family of RE-MOF.

**Figure 1:**
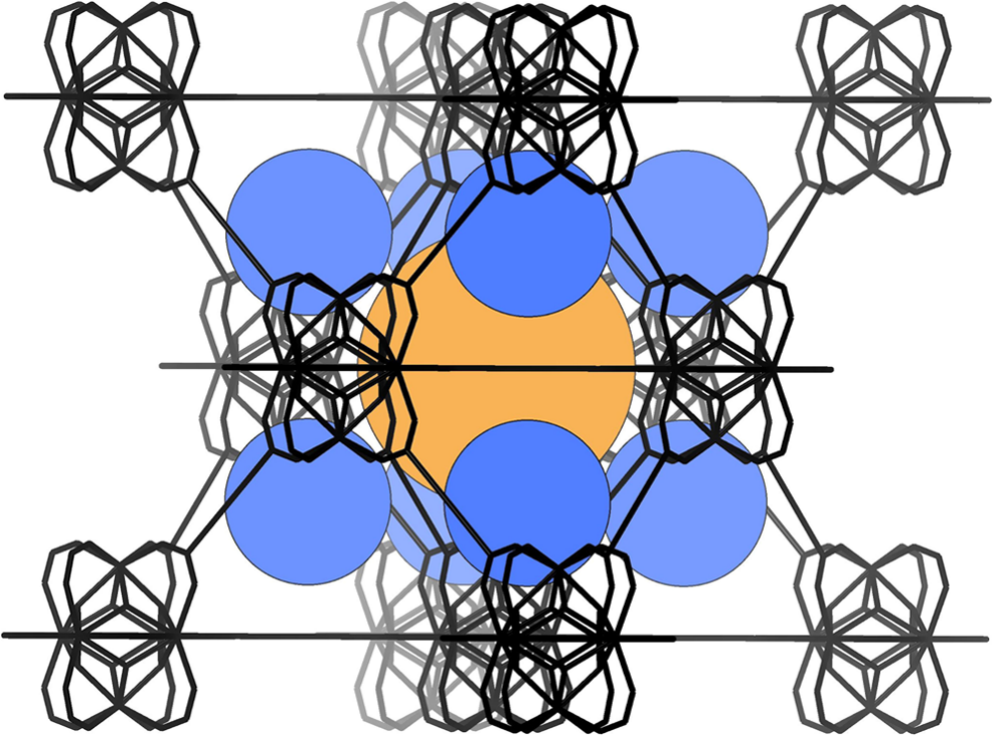
Simplified framework of **1** illustrating the face-centered cubic arrangement of SBUs and the distribution of octahedral and tetrahedral cages. Color key: framework atoms, bonds and simplified organic linker groups = black lines; tetrahedral cages = blue spheres; octahedral cage = orange sphere.

The cuboctahedrally connected RE_6_X_a_R_12_ SBU is structurally equivalent to that found in the significant *fcu* topological MOF group that includes UiO-66 and its numerous derivatives among its members.^17,18^ An archetypal member of this RE-*fcu*-MOF group is Y-ndc-*fcu*-MOF (**1**).^16^ The reported crystallography derived composition of as-synthesized **1** is (DMA)_2_[Y_6_(μ_3_-(OH))_8_(ndc)_6_]·(H_2_O)_6_(solvent)_*x*_ (DMA = dimethylammonium, ndc = 1,4-naphthalenedicarboxylate), with each Y^3+^ ion coordinated by four O atoms from different carboxylate groups, four μ_3_-(OH)^−^ groups, and a water molecule.^16^ The location of the DMA was not determined in the reported crystal structure. The reported framework of **1** consists of a network of face-sharing tetrahedral and octahedral cages which are reachable through three-sided apertures between the cages as shown in Figure 1. **1** and its homologues have been shown to be of particular interest for the selective separation of gas molecules, including to separate carbon dioxide (CO_2_) from dinitrogen (N_2_),^16^ to separate alkane mixtures,^16,19^ to separate hydrogen disulfide and CO_2_ from methane^20^ and to selectively detect ammonia in the presence of other gases.^21^ Surprisingly given the degree of interest in **1**, and other RE-*fcu*-MOFs formed from synthesis mixtures containing modulators or linkers possessing terminal fluorine atoms, there is little direct experimental structural insight concerning the sorption of such guest species within the void space of the framework^12^ and the influence that such species may have on the framework structure.

To these ends, the chemical composition and structure of **1** has been redetermined in the presence of various proportions of a variety of guest species to try to identify and characterize any inherent framework disorder, host–guest interactions and resultant guest-induced framework disorder. We find that the SBU of **1** contains μ_3_-F^–^ groups and that the framework structure undergoes a symmetry changing distortion or different degrees of naphthalene group rotation as a function of the nature of the guest species indicating the subtle framework flexibility of this *fcu*-based system.

## Experimental Methods

### Reagents

Y(NO_3_)_3_·6H_2_O (99.8%, Aldrich) 2-fluorobenzoic acid (97%, Aldrich), H_2_ndc (98+%, Alfa Aesar), anhydrous *N*,*N*-dimethylformamide (DMF) (99.8%, Alfa Aesar), nitric acid (HNO_3_) (70%, Fischer Chemical) and methanol (MeOH) (99.8%, Aldrich) were used as received with no further purification. Distilled water was obtained using a Milli-Q system (18 MΩ cm resistivity at 25 °C).

### Synthesis and Activation of **1**

9.40 mg (0.0435 mmol) H_2_ndc, 48.7 mg (0.348 mmol) 2-fluorbenzoic acid, 2.2 mL anhydrous DMF, 16.7 mg (0.0435 mmol) Y(NO_3_)_3_·6H_2_O, 0.3 mL of 3.5 M HNO_3_ in DMF, and 0.5 mL H_2_O were combined in a 20 mL scintillation vial.^16^ The solution was then sonicated for several minutes to ensure dissolution of the precursors and the sealed vial placed in a preheated oven at 115 °C for 72 h. Once cooled to room temperature, the colorless octahedral crystals of **1 DMF/H**_**2**_**O** were collected by suction filtration, washed with DMF and dried in air.

**1·DMF/H**_**2**_**O** was then solvent exchanged to remove DMF and form MeOH-exchanged **1·MeOH** by covering the sample with 3 mL MeOH twice daily for 5 days. **1·MeOH** was heated in air to 373 K and cooled to room temperature where it adsorbed H_2_O from air to form **1·H**_**2**_**O**.

### Single Crystal X-ray Diffraction

Single crystal X-ray diffraction data collection, data reduction and the experimental equipment used were as previously reported.^12^ All crystal structures were initially solved using the SHELXT^22^ program using an intrinsic phasing method on an hklf4 file implemented through OLEX2 (v1.5),^23^ and refined using the SHELXL^24^ program using least-squares refinement methods against all *F*^2^ values. All non-hydrogen framework atoms were refined with anisotropic displacement parameters or isotropically when the latter was not possible.

Naphth group linker disorder was determined from residual electron density peaks observed after refinement of the ordered component of the structure. The occupancies of the disordered naphth group linker atoms within a particular linker arrangement were fixed or refined against a single free variable assuming that the sum of the occupancies of the linker positions was 1. Geometric restraints were applied to the naphth linker to maintain a suitable linker geometry during refinement. Hydrogen atoms were placed in calculated positions and refined with idealized geometries, assigned fixed occupancies and isotropic displacement parameters. Nonframework species were located, modeled and refined after completion of the framework structure. The nonframework atoms were modeled anisotropically with their occupancies refined against a single free variable when possible. The ordered DMF molecule in **1 DMF/H**_**2**_**O** was modeled by fitting a rigid body model to electron density resembling DMF. The SQUEEZE^25^ procedure was applied to estimate the electrons within the void space for structures where guest species were particularly disordered (**1·DMF/H**_**2**_**O**, **1·MeOH** and **1·N**_**2**_*vide infra*) but was not applied to the CO_2_ containing structures to ensure the most accurate identification and estimation of the electron density associated with the coordinated CO_2_ molecule.

### In Situ Single Crystal X-ray Diffraction Gas Adsorption Studies

**(i) N**_**2(g)**_ A suitable single crystal of **1·H**_**2**_**O** was chosen and activated by heating at 500 K for 2 h before being rapidly cooled (360 K hr^–1^) to 100 K prior to diffraction data collection. The crystal, **1·N**_**2**_, was kept under a stream of dry *N*_2_(*g*) from the cryostream throughout the whole experiment. **(ii) CO**_**2(g)**_ The description of the homemade gas rig and adapted Huber goniometer head “’gas cell’” have been previously reported.^12,26^

A suitable single crystal of **1·MeOH** was selected and activated in situ by heating to 500 K under dry N_2(g)_ followed by fitting the gas cell capillary over the crystal. A vacuum was then applied at 500 K to ensure that no N_2(g)_ was trapped in the capillary and the gas cell was cooled to 298 K. Once the desired temperature of 298 K was reached, 5 bar CO_2(g)_ was then allowed to enter the cell. Data were then collected at 298 and 200 K allowing 30 min equilibrium time at each temperature. The crystal was held at 200 K under 5 bar CO_2_ overnight, and data collected the next morning. During this time, a solid CO_2(s)_ crystal formed at the top of the gas cell capillary. The gas cell and crystal were then warmed to 216 K (the liquid/vapor equilibrium point for 5 bar CO_2_)^27^ and as expected the solid CO_2(s)_ slowly started melting, washing the crystal with liquid CO_2(l)_. After data collection at 216 K, the gas cell was warmed further to 298 K and vacuum applied, and a final data set collected. The structures were reduced, solved and refined against one or two components where nonmerohedral twining was observed. The isotropic atomic displacement parameter for coordinating oxygen atoms of CO_2_ molecules was fixed to 0.1 Å^2^ to avoid atomic displacement parameter-occupancy correlation effects during refinement and to maintain chemically reasonable values of occupancy parameters.

Crystallographic information is provided in Tables S1–S2 and crystallographic information files CCDC 2372655–2372663 contain full details for all crystal structures reported.

### Solid-State NMR Spectroscopy

Magic angle spinning (MAS) NMR spectra were recorded in two regimes. High-field ^19^F MAS NMR spectra were recorded on a Bruker 20.0 T (850 MHz ^1^H Larmor frequency) AVANCE NEO spectrometer equipped with a 1.3 mm HXY MAS probe that was used in ^19^F/^13^C double resonance mode. Experiments were acquired at ambient temperature using a MAS frequency of 60 kHz. ^19^F-pulses of 91 kHz were used, and an echo sequence was employed to reduce interference from the probe background and used a free-evolution delay of 10 rotor periods on either side of the π-pulse, giving a total echo duration of 0.333 ms. Moderate-field ^19^F and ^13^C MAS NMR spectra were recorded on a Bruker 9.4 T (400 MHz ^1^H Larmor frequency) AVANCE III spectrometer equipped with a 4 mm HFX MAS probe that was used in ^1^H/^19^F/^13^C triple resonance mode. Experiments were acquired at ambient temperature using MAS frequencies of 14 and 12 kHz for ^19^F and ^13^C MAS NMR spectra, respectively. ^1^H-, ^19^F-, and ^13^C-pulses of 100, 52, and 50 kHz were used, respectively, and ^13^C NMR spectra were recorded after {^1^H-}^13^C cross-polarization (CP) and echo sequences that used a free-evolution delay of 1 rotor period either side of the π-pulse were employed. For CP, a 70–100% ramp was used for ^1^H (∼73 kHz ν_rf_ at 100%) to match 50 kHz ^13^C spin-locking. ^13^C chemical shifts were referenced to TMS and ^19^F chemical shifts were referenced to CFCl_3_, both at 0 ppm.

### XPS, Elemental Analysis, Powder X-ray Diffraction (PXRD), and Thermogravimetric Analysis (TGA)

Experimental details for XPS, elemental analysis and PXRD are similar to those previously reported.^12^ Le Bail fitting of the PXRD data were performed using GSASII software.^28^ Thermogravimetric analyses were measured on a Mettler-Toledo TGA/DSC instrument under flowing N_2_ from room temperature to 600 °C at a heating rate of 5 °C min^–1^.

## Results and Discussion

### SBU μ_3_-X Group Disorder

The presence of μ_3_-F^–^ groups in the framework of **1** was determined from a variety of techniques. Elemental analyses of phase pure **1·H**_**2**_**O** (see Figure S1) gave a Y:F ratio of 0.77 (Y_6_F_4.6_) indicating the incorporation of fluorine into this previously reported nonfluorine containing MOF. The XPS survey spectrum of evacuated **1·H**_**2**_**O** indicated the occurrence of fluorine and the high-resolution XPS spectra of Y and F identified peaks at 158.7 (Y 3d_5/2_), 160.7 (Y 3d_3/2_) and 685.2 (F) eV respectively (see Figure S2). The values of the Y and F binding energies found for **1** and those reported for Y-fum-*fcu*-MOF,^12^ Y–BCA-3D^13^ and RE-frt-MOF-1^29^ suggest very similar chemical states for these atoms in these MOFs that all contain a (Y_6_F_a_) (*a* ≤ 8) core within the SBU and μ_3_-F^–^ groups. ^19^F magic angle spinning (MAS) NMR spectra of **1·DMF/H**_**2**_**O** and **1·MeOH** show a single peak with chemical shift δ{^19^F} = −65 ppm (see Figure S3a) indicating the presence of only one F species in the Y_6_F_4.6_ core of the SBU. This shift matches those reported for the μ_3_-F^–^ group of this SBU type present in other RE-F-*fcu*-MOFs, namely Y-fum-*fcu*-MOF^12^ and Y-DOBDC MOF.^14^ The presence of μ_3_-F^–^ groups in the Y_6_(μ_3_-X)_a_ core of the SBU is also supported by the X-ray diffraction determined crystal structures of **1** in the presence of different sorbates (*vide infra*). The atomic displacement parameters of the μ_3_-groups within the crystal structures at 100 K for nonthermally activated samples are most consistent with the presence of F atoms with 100% site occupancy (see Table S3 for **1·DMF/H**_**2**_**O**), implying a majority of face-capping μ_3_-F^–^ sites in the Y_6_(μ_3_-X)_a_ core (X = F^–^, (OH)^−^ or O^2–^, *a* ≤ 8) supporting the above results. Hence, the μ_3_-X sites in the subsequent sections are named for simplicity in terms of the major μ_3_-X species, F. These results demonstrate that the SBU of **1** contains a majority of μ_3_-F^–^ groups disordered over the μ_3_-X positions and provides another example of a RE-MOF for which the fluoro-containing modulator molecule provides fluoride ions to the SBU.^1^ The amount of F^–^ incorporated into the SBU (Y_6_F_4.6_) is less than that reported in Y-fum-*fcu*-MOF (Y_6_F_5.6_),^12^ and Y-DOBDC MOF (Y_6_F_∼6_)^14^ that both possess the same type of SBU. This demonstrates that a variable degree of fluoride inclusion occurs that is presumably dependent on the synthetic conditions, for example reagents, pH, temperature, and pressure, employed to prepare the RE-MOF as examined by Balkus et al.^30^ The degree of fluoride inclusion is a critical parameter to control as it has potentially important ramifications on the properties of the resultant MOFs including those of hydrophobicity, photoluminescence and guest sorption.^1,12^

### Guest-Induced Framework Phase Transitions

#### **1·DMF/H_2_O**

The crystal structure of **1·DMF/H**_**2**_**O** was solved and refined in the cubic *Pa*3̅ space group instead of the previously reported cubic *Fm*3̅*m* space group^16^ after initial inspection of the diffraction data showed the presence of weak diffraction spots that break the F-centering of the pattern and consideration of observed reflection condition data (as shown in Figure S4a–b and given in Tables S4–S7). The Le Bail fit using the PXRD data collected from a bulk powder sample of **1·DMF/H**_**2**_**O** shown in Figure S5 supports this space group assignment. The crystallographically determined framework formula of **1·DMF/H**_**2**_**O** is [Y_6_(μ_3_-F)_8_(ndc)_6_]^2–^·(DMF)_1.38(6)_(H_2_O)_1.7(1)_ where framework interacting guest molecules have not been included as part of the framework formula and the charge balancing DMF-derived DMA ions were not located. The presence of DMA and DMF in **1·DMF/H**_**2**_**O** are seen in the ^13^C MAS NMR spectrum as shown in Figure S3b.

**Figure 2:**
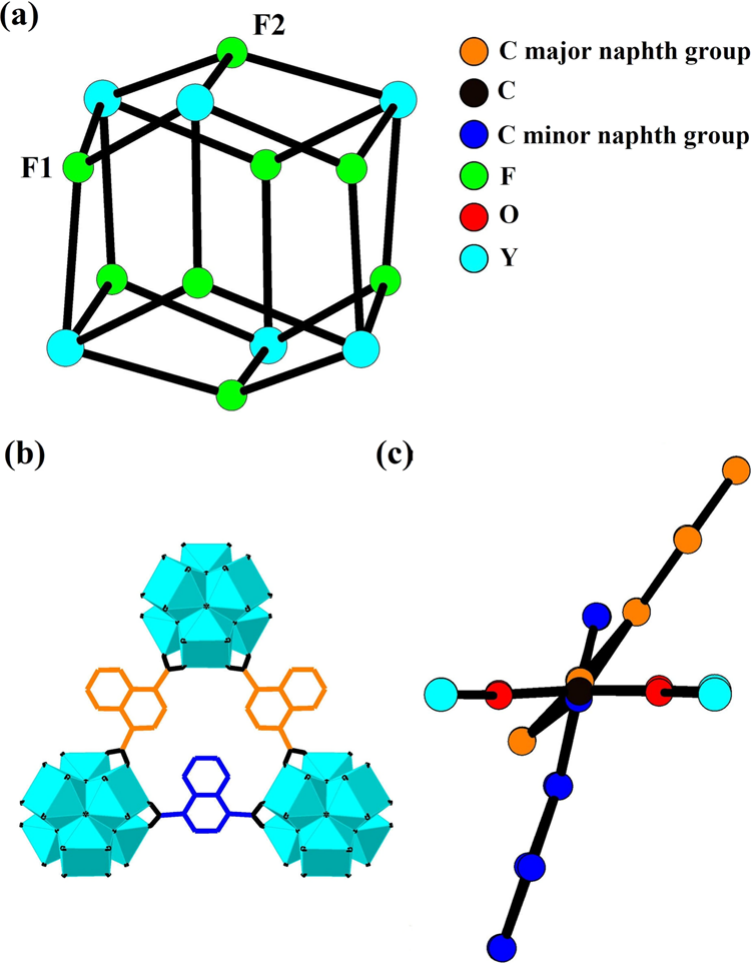
Representations of the Y_6_F_8_ core of the SBU (a), a triangular aperture found within the framework showing the two crystallographically distinct naphth linker groups (b) and the two possible crystallographically distinct naphth linker groups shown at one linker position viewed along the axis formed through the C atoms of the two carboxylate groups of the linker (c) in **1·DMF/H**_**2**_**O**. H atoms are omitted for clarity.

The Y_6_ core of the SBU possesses trigonal antiprismatic geometry with two equilateral and six isosceles triangular faces. The isosceles and equilateral triangular faces are capped by the two crystallographically distinct μ_3_-F^–^ sites, F1 and F2, respectively as seen in Figure 2a. The framework of **1·DMF/H**_**2**_**O** contains one type of octahedral and tetrahedral cage with the latter being lined internally with three F1 and one F2 ions at their vertices. Despite the crystallographic observation of two distinct μ_3_-F^–^ sites, a single Lorentzian peak is found in the ^19^F MAS NMR spectrum of **1·DMF/H**_**2**_**O** (and at high field for **1·MeOH**, *vide supra*). The presence of a single peak only was attributed to insufficient ^19^F NMR spectral resolution to resolve the signals from the two distinct μ_3_-F^–^ sites with nearly identical chemical environments. This is in contrast to the distinguishable μ_3_-F^–^ sites of Y-fum-*fcu*-MOF,^12^ which results from the different local environments of the two μ_3_-F^–^ sites with regards to the distinct orientations of the fumerate linker in the framework.

**Figure 3:**
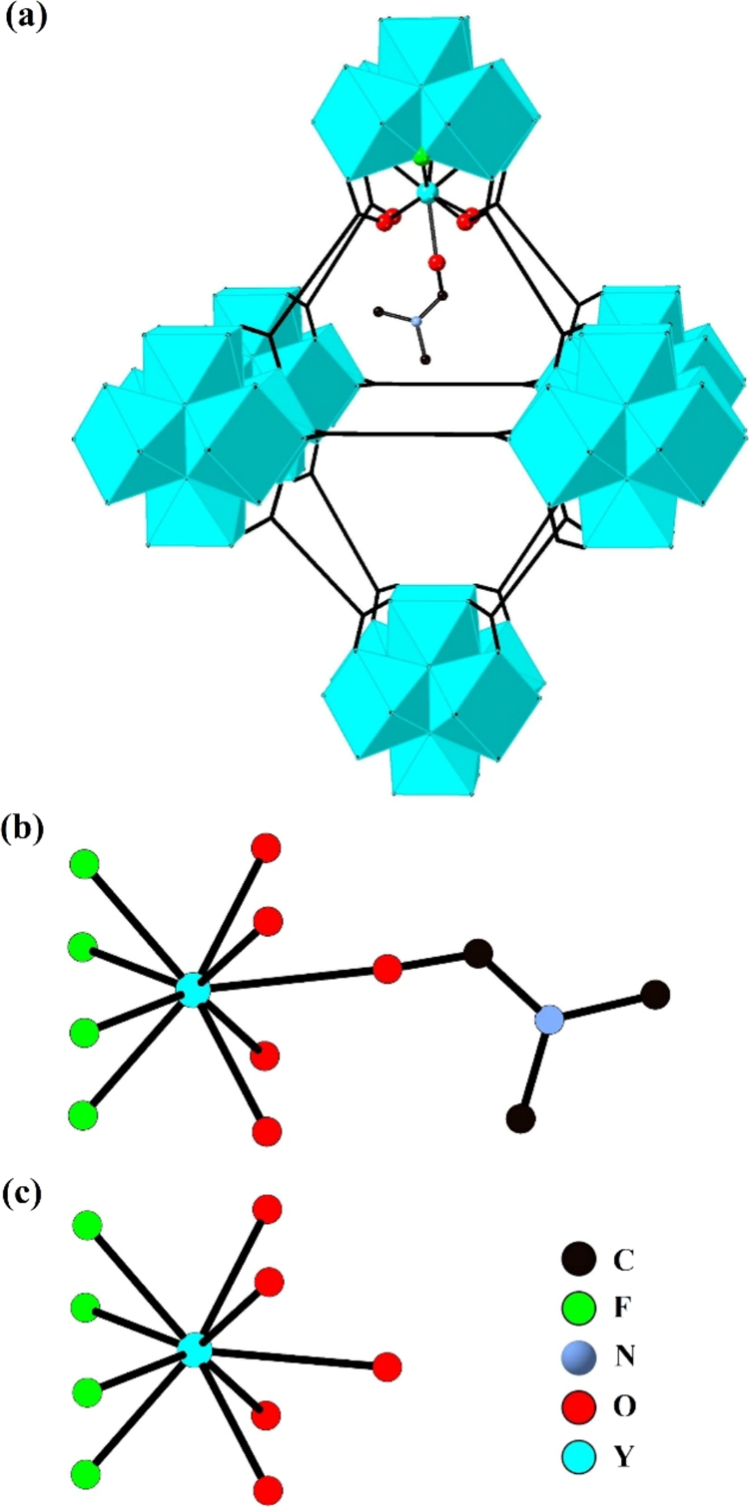
Representations of the coordination of a DMF molecule to a Y center within a simplified octahedral framework cage (a) and in greater detail at a Y center (b) and the coordination of a H_2_O molecule to a Y center (c) in **1·DMF/H**_**2**_**O**. H atoms are omitted for clarity.

The framework of **1·DMF/H**_**2**_**O** contains disordered naphth groups of the ndc linker. The naphth linker groups are disordered over two crystallographically distinct orientations. The major naphth orientation has an occupancy of 77.7(4) % and the minor component has an occupancy of 22.3(4) %, as shown in Figure 2b,c. Framework coordinated DMF and H_2_O molecules were located within the octahedral cages where the electron rich oxygen atoms of both molecules interact with the electron deficient Y^3+^ ions as shown in Figure 3a–c. Both molecules are relatively weakly coordinated with Y–O bond lengths of 2.61(1) and 2.60(2) Å for DMF and H_2_O respectively. The occupancy of the DMF and H_2_O molecules are 23(1) and 28(1) % respectively, occupying 51(2) % of the available open Y sites.

The ordered nature of the major naphth orientation component, in comparison to the symmetry disordered naphth linker in the previously reported *Fm*3̅*m* structure and described *vide infra*,^16^ most likely results from interactions between the naphth group and disordered guest molecules present in the void volume such as DMF, H_2_O or DMA ions, with the DMF molecules most likely to be instrumental in the ordering of the naphth groups due partially to their larger size. The amount of the minor naphth orientation component (22.3(4)%) appears to precisely correlate with the occupancy of the framework-bound DMF molecules (23(1) %) implying that the localized DMF molecules lead to the adoption of the minor naphth component orientation and the reduction of symmetry to the cubic *Pa*3̅ space group. There are no obvious favorable or unfavorable intermolecular interactions between the framework coordinated DMF molecules and the naphth moieties suggesting that the steric effect induced by the presence of the coordinated DMF molecules influence the orientation of the naphth groups in the ndc linker.

#### **1·MeOH**

**Figure 4:**
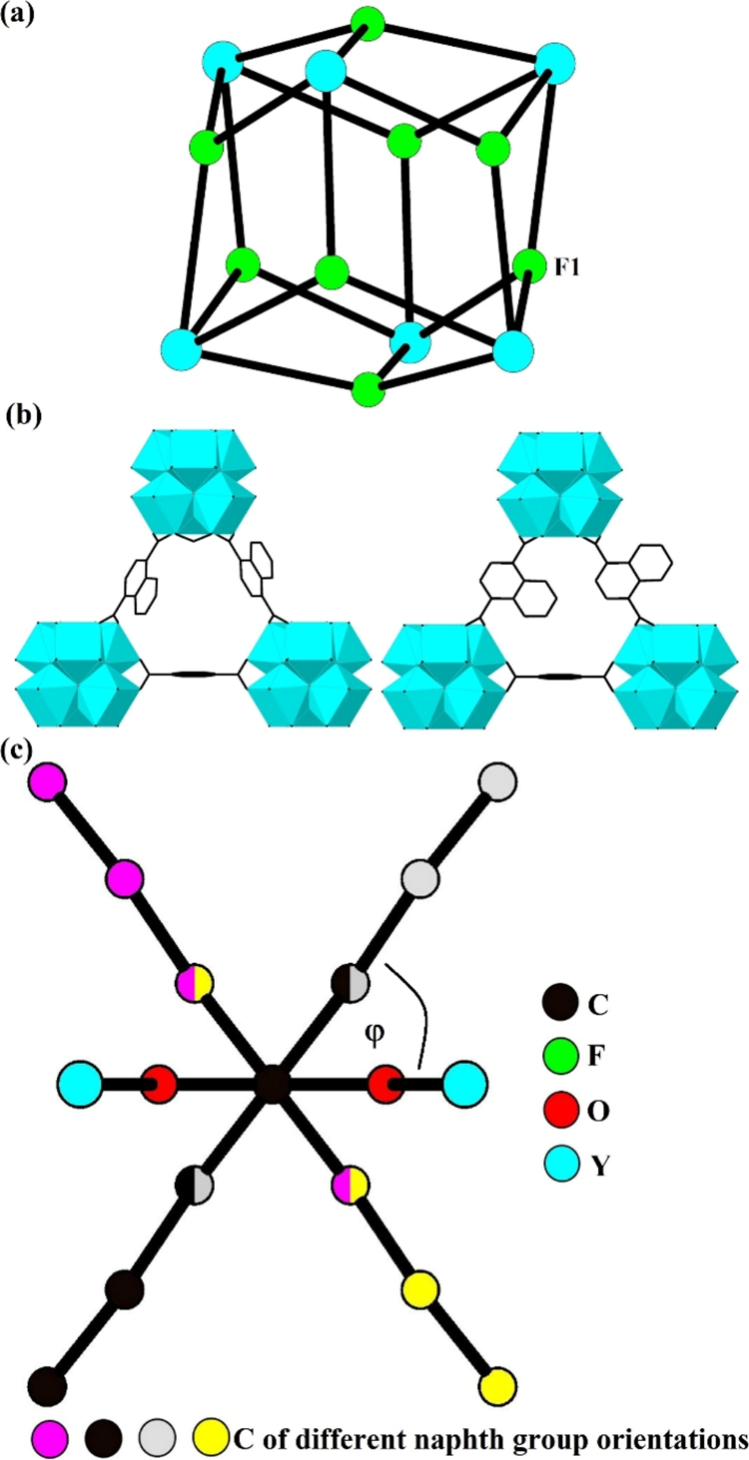
Representations of the Y_6_F_8_ core of the SBU (a), triangular apertures found within the framework showing the four possible symmetry related orientations of the naphth linker groups (b) and the four possible symmetry related orientations of the naphth groups of the crystallographically unique ndc linker shown at one framework linker position viewed along the axis formed through the C atoms of the two carboxylate groups of the linker (c) in **1·MeOH**. Split occupancy atom sites are represented with two colors in (c) and H atoms are omitted for clarity.

The TGA and PXRD results for **1·MeOH** shown in Figure S7 and S6 show marked differences to those for **1·DMF/H**_**2**_**O** indicating successful solvent exchange of DMF for MeOH. The crystal structure of **1·MeOH** was solved and refined in the cubic *Fm*3̅*m* space group as no weak F-symmetry breaking diffraction peaks were observed in the diffraction data (see Table S7). The Le Bail fit using the PXRD data collected from a bulk powder sample of **1·MeOH** shown in Figure S6 again supports this space group assignment. The crystallographically determined framework formula of **1·MeOH** is [Y_6_(μ_3_-F)_8_(ndc)_6_]^2–^·(MeOH)_1.8(1)_ where the charge balancing DMA ions were not located. The Y_6_ core of the SBU possesses octahedral geometry with the eight equilateral faces capped by the μ_3_-F^–^ crystallographically distinct F1 site as seen in Figure 4a. The framework of **1·MeOH** contains one type of octahedral and tetrahedral cage with the latter being lined internally with four F1 ions at their vertices.

**Figure 5:**
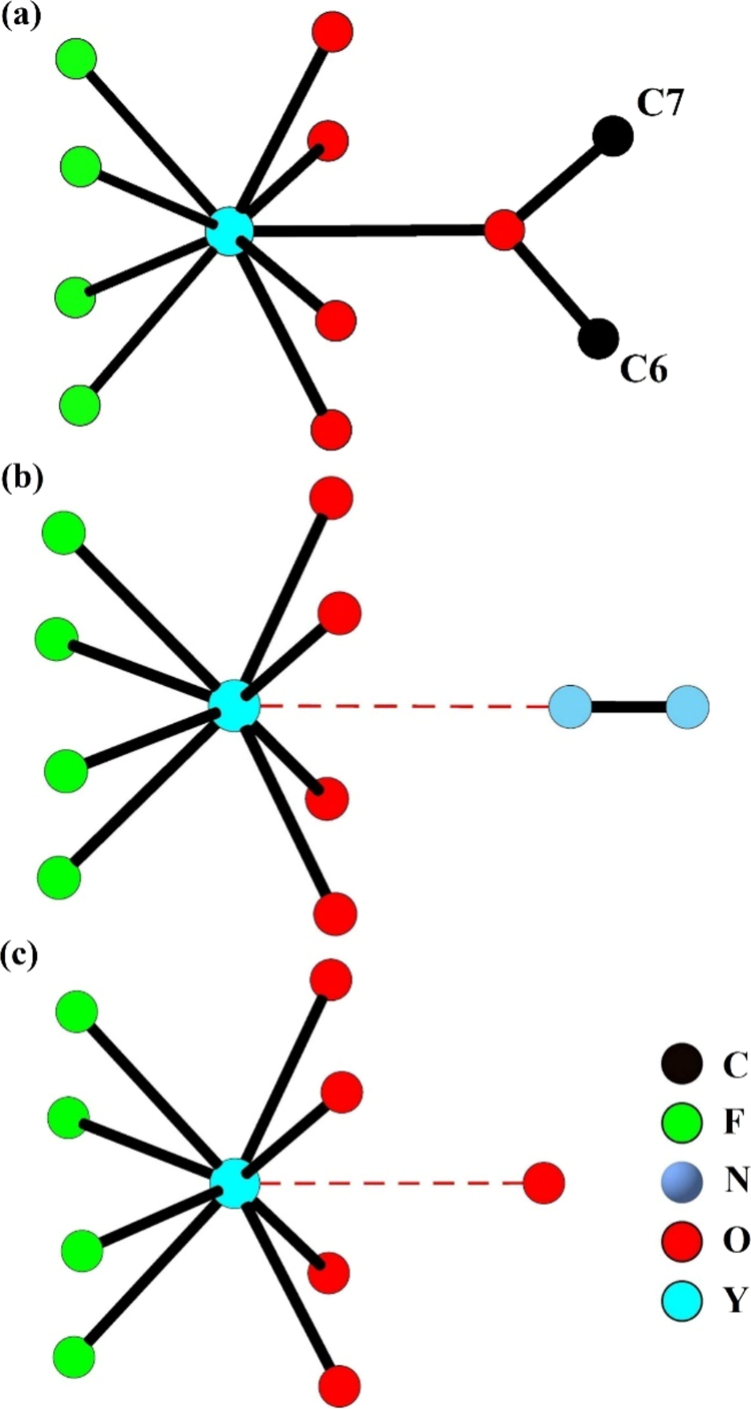
Representations of the coordination of the two crystallographically distinct MeOH molecules to a Y center in **1·MeOH** (a) the closest interaction of a N_2_ molecule to a Y center in **1·N**_**2**_ (b) and the closest interaction of the end O atom of a CO_2_ molecule to a Y center in **1·CO**_**2**_**-200 K** (c). Selected closest interaction distances are shown as red dotted lines and H atoms are omitted for clarity.

The framework of **1·MeOH** contains disordered naphth groups of the linker over four symmetry related orientations as shown in Figure 4b–c, and as previously reported.^16^ Framework coordinated MeOH molecules were located within the octahedral cages where the electron rich oxygen atoms of the MeOH molecules interact with the electron deficient Y^3+^ ions as shown in Figure 5a. Eight equally probable orientations of the MeOH molecules were located for which the MeOH molecules are coordinated with Y–O_MeOH_-C_MeOH_ angles of 129(3) (for C6) or 137(3) ° (for C7) relative to the Y site. The MeOH is relatively weakly coordinated with a Y–O bond length of 2.64(2) Å and occupies 30(2) % of the available open Y sites. The high degree of disorder of the naphth group and MeOH suggests that interaction between the two is negligible and the presence of MeOH as the guest does not reduce the symmetry of the structure from the *Fm*3̅*m* space group due in part to its small molecular size compared to DMF.

#### **1·N_2_**

The crystal structure of **1·N**_**2**_ was solved and refined in the cubic *Fm*3̅*m* space group. The crystallographically determined framework formula of **1·N**_**2**_ is [Y_6_(μ_3_-F)_8_(ndc)_6_]^2–^·(N_2_)_3.2(2)_ where the charge balancing DMA ions were not located. The framework of **1·N**_**2**_ is like that of **1·MeOH** with an octahedral Y_6_ core of the SBU and the disorder of the naphth groups of the linker over four symmetry related orientations in a similar manner to that shown in Figure 4a–c, respectively. Framework coordinated N_2_ molecules were located within the octahedral cages where the electron rich nitrogen atoms of the N_2_ molecules interact with the Lewis acidic Y^3+^ centers and the N_2_ molecule is arranged linearly with respect to the Y^3+^ center as shown in Figure 5b. The refined atomic nitrogen positions gave a N–N distance of 1.05(4) Å matching that expected within the triply bonded N_2_.^31,32^ The N_2_ is weakly coordinated as indicated by the long Y···N distance of 3.03(3) Å and occupies 53(3) % of the available open Y sites. The reported N_2_ BET isotherm for **1**(16) shows significant adsorption at a relative pressure (P/P^o^) of ∼0.15 corresponding well with the physisorption observed here at 100 K (P/P^o^ = 0.128, assuming 1 bar N_2_). Similar low pressure physisorption behavior of *N*_2_(*g*) has also been observed for Y-fum-*fcu*-MOF.^12^ Again, the high degree of disorder of the naphth group suggests that the interaction between the N_2_ and linker is negligible and the linear nature of the small N_2_ molecule does not induce a reduction of the symmetry from the *Fm*3̅*m* space group for **1·N**_**2**_.

#### In Situ CO_2_ Adsorption

A summary of the conditions and crystallographic results for **1** obtained from the in situ CO_2_ adsorption experiment are presented in Table 1. The crystal structures of **1** with sequence numbers 1–6 in Table 1 were solved and refined in the cubic *Fm*3̅*m* space group with similar frameworks to that described for **1·MeOH** and **1·N**_**2**_ (*vide supra*) and shown in Figure 4a–c. The nonframework DMA ions and CO_2_ molecules were not located, however electron density at ∼2.9 Å from the Y^3+^ centers was observed after CO_2_ was admitted to the crystal. This electron density was modeled as the coordinating oxygen atom of a CO_2_ molecule as shown in Figure 5c and was located at a distance in the range 2.80(4) – 2.96(2) Å within **1** at the different points in the in situ experiment, indicating that CO_2_ molecules interact with the Y^3+^ centers with strength intermediate between H_2_O/DMF/MeOH and N_2_.

**Table 1:** Crystallographic Occupancy of the Coordinating O Atom of a CO_2_ Molecule within 1 and the Unit Cell Volume of 1 under Different Applied Conditions during an In Situ CO_2_ Gas Adsorption Experiment

sequence number and sample name	conditions	CO_2_ P/P^o^	coordinating O atom occupancy	unit cell volume (Å^**3**^)
1	vacuum, 500 K, <5 mbar	0	0	9607(1)
**1·vac-500 K**				
2	5 bar CO_2_, 298 K	0.077	0.40(2)	9783(1)
**1·CO_2_-298 K**				
3	5 bar CO_2_, 200 K	>1	0.82(3)	9928.8(3)
**1·CO_2_-200 K**				
4	5 bar CO_2_, 200 K, overnight	>1	0.86(3)	9866(1)
**1·CO**_**2**_**-200 K-overnight**				
5	5 bar CO_2_, 216 K	0.99	0.76(2)	9798.8(4)
**1·CO_2_-216 K**				
6	vacuum, <5 mbar, 298 K	0	0.39(3)	9720.1(3)
**1·vac-298 K**				

The occupancy of the coordinating O atom of the CO_2_ increases as the temperature at which the crystal is held decreases with an accompanying increase in unit cell volume for the higher occupancies as seen in Table 1. On increasing the temperature from 200 to 216 K at 5 bar CO_2_ pressure the accompanying change from solid to liquid CO_2_ within the gas cell capillary causes nonmerohedral twinning of the crystal presumably due to reorientation of part of the crystal during desorption with possible accompanying strain relief. This nonmerohedral twinning persists during subsequent reheating of the crystal to 298 K and exposure to vacuum for 30 min. Significant electron density was observed at 2.7(1) Å from the Y^3+^ atoms in **1·vac-298 K** suggesting that not all CO_2_ or potentially other residual guest molecules were removed during this treatment.

The location of the coordinating O atom of CO_2_ is similar to that found in Y-fum-*fcu*-MOF^12^ and that suggested in the closely related MOF (DMA)_2_[Tb_6_[μ_3_-(OH)]_8_(ftzb)_6_(H_2_O)_6_] (ftzb = 2-fluoro-4-(1H-tetrazol-5yl)benzoate) where the extension of the CO_2_ molecules into the octahedral cage is stabilized by additional interactions with the linkers.^11^ The favored position of CO_2_ within **1**, Y-fum-*fcu*-MOF and (DMA)_2_[Tb_6_[μ_3_-(OH)]_8_(ftzb)_6_(H_2_O)_6_] contrast with the location of CO_2_ molecules within UiO-66 where the adsorbed CO_2_ molecules are found in the tetrahedral cages of the framework interacting with the μ_3_–OH groups.^33^ Again, the high degree of disorder of the naphth group suggests that the interaction between the CO_2_ and linker is negligible and the linear nature of the small CO_2_ molecule does not induce a reduction of the symmetry from the *Fm*3̅*m* space group.

**Scheme 1:**
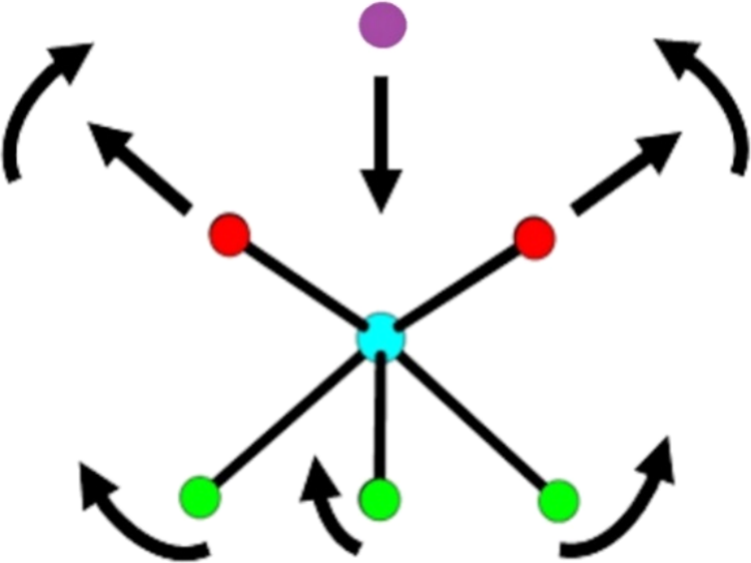
Schematic of the Motion of Various Atoms in the YO_4_F_4_ Polyhedron as an Atom of a Guest Molecule Approaches the Y^3+^ Center Color key: Y = cyan, F = green, O = red, atom from guest = purple.

Consideration of this set of host–guest compounds and the structure of the framework of **1** in the presence of different types of guest molecule reveal certain aspects of structural flexibility within the SBU and linker components of the *fcu* framework. The SBU of **1** is found to subtly flex when different guest molecules interact with the open Y^3+^ centers with the strongest interaction causing larger distortion of the Y-centered square antiprism. This is exemplified in the geometric parameters presented in Table 2 and Scheme 1, where the greater the interaction of the guest molecule with the Y^3+^ center, the greater the Y–O_ndc_ bond length and narrower the O_ndc_–Y-O_ndc_ angles. Simultaneously, little happens to the Y–F bond length but there is a noticeable expansion in all the F–Y–F-angles. Small changes occur for the O_ndc_–Y-F angles as the interaction with the guest molecule increases. The presence of guest species in the void space of **1** also lead to a guest-induced framework distortion resulting primarily from different orientations, and for the *P*a3̅ structure, positions of the naphth groups of the linker. These orientations are shown in Figures 2b–c and 4b–c and quantified by the torsion angle (φ) between the naphth and the carboxylate groups of each ndc linker given in Table 3. The variation in φ with the size and orientation of the guest species relative to the Y^3+^ center suggest that nonlinear guest molecules that coordinate to the Y^3+^ center in a nonlinear manner appear to cause the greater rotation of the naphth group from the plane of the associated carboxylate groups, most noticeably for the **1·DMF/H**_**2**_**O** minor naphth group orientation. However, further correlations are difficult to determine due to the large number of positionally undetermined molecules in the void space of **1** within these host–guest compounds.

**Table 2:** Geometric Parameters of a YO_4_F_4_ Square Antiprism as It Interacts with Various Guest Molecules at 100 K

sample	Y–O/N_guest_ distance (Å)	Y–O_ndc_ distance (Å)	Y–F distance (Å)	*cis* -O_ndc_–Y-O_ndc_ angle (deg)	*cis*-O_ndc_–Y-F angle (deg)	*cis*–F–Y-F angle (deg)	*trans*-O_ndc_–Y-O_ndc_ angle (deg)	*trans*-O_ndc_–Y-F angle (deg)	*trans-*F–Y-F angle (deg)
**1·N**_**2**_	3.03(3)	2.257(3)	2.302(2)	80.88(7)	78.2(2)	62.4(2)	133.1(2)	138.27(7)	94.3(3)
**1·MeOH**	2.64(2)	2.313(3)	2.296(1)	79.61(7)	76.9(1)	66.3(1)	129.7(2)	139.85(5)	101.4(2)
**1·DMF/H_2_O**^a^	2.61(2)	2.314(3)	2.293(1)	79.74(6)	76.87(8)	66.22(7)	130.1(1)	139.75(5)	101.2(1)

a Averaged geometric parameters.

**Table 3:** Torsion Angle (φ) between the Naphth Group and the Carboxylate Group of an Ndc Linker as Defined in Figure S8 and Shown in Figure 4c

sample	φ (deg)
**1·DMF/H_2_O** major naphth orientation	46.2(6), 47.3(9)
**1·DMF/H**_**2**_**O** minor naphth orientation	72(2), 72(4)
**1·MeOH**	51.7(7)
**1·N**_**2**_	43.3(6)
**1·CO_2_-200 K**	45.3(7)
**1·CO_2_-200 K-overnight**	42(1)
**1·CO**_**2**_**-216 K**	41(1)

## Conclusions

This work provides another example of a RE-MOF, prepared in the presence of a modulator molecule possessing a terminal fluorine atom, that contains μ_3_-face-capping F^–^ groups within its SBU suggesting that many of the already reported RE-MOFs synthesized with this protocol might contain μ_3_-F^–^ groups.^1^ It is also apparent that the degree of incorporation of μ_3_-F^–^ groups within the same Y_6_F_a_ (*a* ≤ 8) core in the SBU unit is dependent on the reagents and conditions utilized during synthesis of the MOF. This indicates that it should be possible to tailor the composition of the Y_6_F_a_ (*a* ≤ 8) core in this SBU in a chemically controlled manner for a desired functional application as has been shown possible for other cores in RE-MOFs.^34^ The most favored location of guest molecules containing electron rich atoms is found to be within the octahedral cages of the **1** where they interact most strongly with the Lewis acidic Y^3+^ centers. The size, shape, and interactions of the guest species leads to subtle distortions within the SBU and specific orientations of the linker naphth group within the framework of **1**. This demonstrates that the framework of a MOF is a relatively flexible entity that responsively adapts to the type of guest molecule and that such guest-induced framework transitions may be an important consideration in both understanding and optimizing the performance of these compounds during commercially relevant applications.

## Supporting Information Available

Additional structural and diffraction information, crystallographic information files CCDC 2372655–2372663, XPS spectra, NMR spectra and TGA traces (PDF)
