# Supplementary material for: Revealing Disorder, Sorption Locations and a Sorption-Induced Single Crystal–Single Crystal Transformation in a Rare-Earth fcu-Type Metal–Organic Framework
Source: Inorg Chem. 2024 Nov 4;63(46):22315–22. doi: 10.1021/acs.inorgchem.4c04286 (PMC11577313; doi:10.1021/acs.inorgchem.4c04286)
Supplement: Supplementary file 1 — ic4c04286_si_001.pdf [file ic4c04286_si_001.pdf]

Supporting Information

Revealing Disorder, Sorption Locations and a Sorption Induced Single Crystal-Single Crystal Transformation in a Rare Earth *fcu*-Type Metal-Organic Framework

A. R. Bonity J. Lutton-Gething,[a#] Fajar I. Pambudi,[a§] Ben F. Spencer,[b,c] Daniel Lee,[d] George F. S. Whitehead,[a] Inigo J. Vitorica-Yrezabal,[a†] and Martin P. Attfield[a*]

[a]Department of Chemistry, School of Natural Sciences, The University of Manchester, Oxford Road, Manchester, M13 9PL, U.K.

[b]Department of Materials and National Graphene Institute, The University of Manchester, Oxford Road, Manchester, M13 9PL, U.K.

[c]Photon Science Institute, The University of Manchester, Oxford Road, Manchester, M13 9PL, U.K.

[d]Department of Chemical Engineering, School of Engineering, The University of Manchester, Oxford Road, Manchester, M13 9PL, U.K

#School of Chemistry, The University of Birmingham, Edgbaston, Birmingham, B15 2TT, U.K.
§Department of Chemistry, Universitas Gadjah Mada, Sekip Utara, Yogyakarta, 55281, Indonesia.
†Fuente Nueva SN, Facultad de Ciencias, Departamento de Quimica Inorganica Universidad de Granada, Granada, 18071, Spain

*Corresponding author, email: m.attfield@manchester.ac.uk

Table S1: Summary of crystal data and refinement details for 1·DMF/H_2O, 1·MeOH and 1·N_2

Crystal Structure	1·DMF/H_2O	1·MeOH	1·N_2
CCDC number	2372661	2372662	2372663
Empirical formula	$C_{76.14}H_{49.01}F_8N_{1.38}O_{27.06}Y_6$	$C_{79.46}H_{24}F_8O_{25.86}Y_6$	$C_{72}H_{24}F_8N_{6.34}O_{24}Y_6$
Formula weight	2101.52	2077.78	2047.15
Temperature/K	100.00(11)	100.00(13)	99.9(8)
Crystal system	cubic	cubic	cubic
Space group	Pa-3	Fm-3m	Fm-3m
a/Å	21.3829(2)	21.3637(2)	21.26700(10)
Volume/Å^3	9776.9(3)	9750.6(3)	9618.75(14)
Z	4	4	4
ρ_{calc}g/cm^3	1.428	1.415	1.414
μ/mm^{-1}	5.283	5.284	5.351
F(000)	4152.0	4055.0	3993.0
Crystal size/mm^3	0.09 × 0.077 × 0.056	0.092 × 0.072 × 0.058	0.6 × 0.25 × 0.09
Radiation	Cu Kα (λ = 1.54184)	Cu Kα (λ = 1.54184)	Cu Kα (λ = 1.54184)
2Θ range for data collection/°	7.16 to 152.22	7.166 to 151.998	8.316 to 151.848
Index ranges	-26 ≤ h ≤ 24, -20 ≤ k ≤ 26, -17 ≤ l ≤ 20	-26 ≤ h ≤ 17, -24 ≤ k ≤ 9, -9 ≤ l ≤ 24	-19 ≤ h ≤ 26, -20 ≤ k ≤ 21, -23 ≤ l ≤ 19
Reflections collected	12945	3126	3260
Independent reflections	3321 [R_{int} = 0.0143, R_{sigma} = 0.0130]	558 [R_{int} = 0.0112, R_{sigma} = 0.0048]	538 [R_{int} = 0.0130, R_{sigma} = 0.0090]
Data/restraints/parameters	3321/510/293	558/73/58	538/57/55
Goodness-of-fit on F^2	1.127	1.208	1.122
Final R indexes [I>=2σ (I)]	R_1 = 0.0604, wR_2 = 0.2184	R_1 = 0.0366, wR_2 = 0.1144	R_1 = 0.0379, wR_2 = 0.1061
Final R indexes [all data]	R_1 = 0.0649, wR_2 = 0.2282	R_1 = 0.0367, wR_2 = 0.1145	R_1 = 0.0382, wR_2 = 0.1062
Largest diff. peak/hole / e Å^{-3}	3.52/-0.71	0.66/-0.50	0.36/-0.42

Table S2: Summary of crystal data and refinement details for the crystal structure of 1 determined during an in situ single crystal X-ray diffraction CO_2 gas adsorption experiment

Sequence Number/conditions	1. 1·vac-500K	2. 1·CO_2-298K	3. 1-CO_2-200K	4. 1-CO_2-200K-overnight	5. 1-CO_2-216K	6. 1-vac-298K
CCDC number	2372655	2372656	2372657	2372658	2372659	2372660
Empirical formula	$C_{72}H_{24}F_8O_{24}Y_6$	$C_{72}H_{24}F_8O_{26.46}Y_6$	$C_{72}H_{36}F_8O_{28.92}Y_6$	$C_{72}H_{24}F_8O_{29.2}Y_6$	$C_{72}H_{36}F_8O_{28.38}Y_6$	$C_{72}H_{24}F_8O_{26.4}Y_6$
Formula weight	1958.37	1997.45	2049.19	2041.53	2040.47	1996.81
Temperature/K	500.00(10)	298.01(10)	200.00(10)	200.00(10)	216.00(10)	298.01(10)
Crystal system	cubic	cubic	cubic	cubic	cubic	cubic
Space group	Fm-3m	Fm-3m	Fm-3m	Fm-3m	Fm-3m	Fm-3m
a/Å	21.2584(8)	21.3876(8)	21.4931(2)	21.4476(8)	21.3989(3)	21.3414(2)
Volume/Å^3	9607.1(11)	9783.3(11)	9928.8(3)	9865.9(11)	9798.8(4)	9720.1(3)
Z	4	4	4	4	4	4
ρ_{calc}g/cm^3	1.354	1.356	1.371	1.374	1.383	1.365
μ/mm^{-1}	3.657	3.594	3.545	3.568	5.262	5.280
F(000)	3816.0	3894.0	4021.0	3982.0	4004.0	3893.0
Crystal size/mm^3	0.09 × 0.07 × 0.06	0.09 × 0.07 × 0.06	0.09 × 0.07 × 0.06	0.09 × 0.07 × 0.06	0.09 × 0.07 × 0.06	0.09 × 0.07 × 0.06
Radiation	Mo Kα (λ = 0.71073)	Mo Kα (λ = 0.71073)	Mo Kα (λ = 0.71073)	Mo Kα (λ = 0.71073)	Cu Kα (λ = 1.54184)	Cu Kα (λ = 1.54184)
2Θ range for data collection/°	3.318 to 56.74	3.808 to 60.526	3.79 to 56.48	3.798 to 59.904	7.156 to 149.156	7.174 to 150.296
Index ranges	-10 ≤ h ≤ 24, -27 ≤ k ≤ 18, -24 ≤ l ≤ 25	-19 ≤ h ≤ 21, -18 ≤ k ≤ 5, -26 ≤ l ≤ 17	-12 ≤ h ≤ 25, -19 ≤ k ≤ 28, -26 ≤ l ≤ 24	-29 ≤ h ≤ 17, -27 ≤ k ≤ 22, -10 ≤ l ≤ 24	-21 ≤ h ≤ 26, -26 ≤ k ≤ 26, -22 ≤ l ≤ 23	-21 ≤ h ≤ 25, -20 ≤ k ≤ 24, -24 ≤ l ≤ 26
Reflections collected	3370	3527	4366	3472	1791	6832
Independent reflections	581 [R_{int} = 0.0638, R_{sigma} = 0.0621]	671 [R_{int} = 0.0379, R_{sigma} = 0.0350]	604 [R_{int} = 0.0728, R_{sigma} = 0.0523]	682 [R_{int} = 0.0383, R_{sigma} = 0.0362]	1791 [R_{int} = 0.0447, R_{sigma} = 0.0140]	563 [R_{int} = 0.0238, R_{sigma} = 0.0096]
Data/restraints/parameters	581/59/48	671/70/50	604/60/50	682/67/50	1791/60/51	563/53/50
Goodness-of-fit on F^2	1.082	1.120	1.148	1.165	1.264	1.347
Final R indexes [I>=2σ (I)]	R_1 = 0.0478, wR_2 = 0.1236	R_1 = 0.0543, wR_2 = 0.1512	R_1 = 0.0739, wR_2 = 0.2208	R_1 = 0.0639, wR_2 = 0.2052	R_1 = 0.0695, wR_2 = 0.2341	R_1 = 0.0529, wR_2 = 0.1570
Final R indexes [all data]	R_1 = 0.0650, wR_2 = 0.1329	R_1 = 0.0743, wR_2 = 0.1607	R_1 = 0.0931, wR_2 = 0.2322	R_1 = 0.0797, wR_2 = 0.2174	R_1 = 0.0741, wR_2 = 0.2416	R_1 = 0.0534, wR_2 = 0.1575
Largest diff. peak/hole / e Å^{-3}	0.45/-0.54	0.54/-0.48	0.70/-1.24	0.80/-1.61	1.39/-1.09	1.12/-0.83

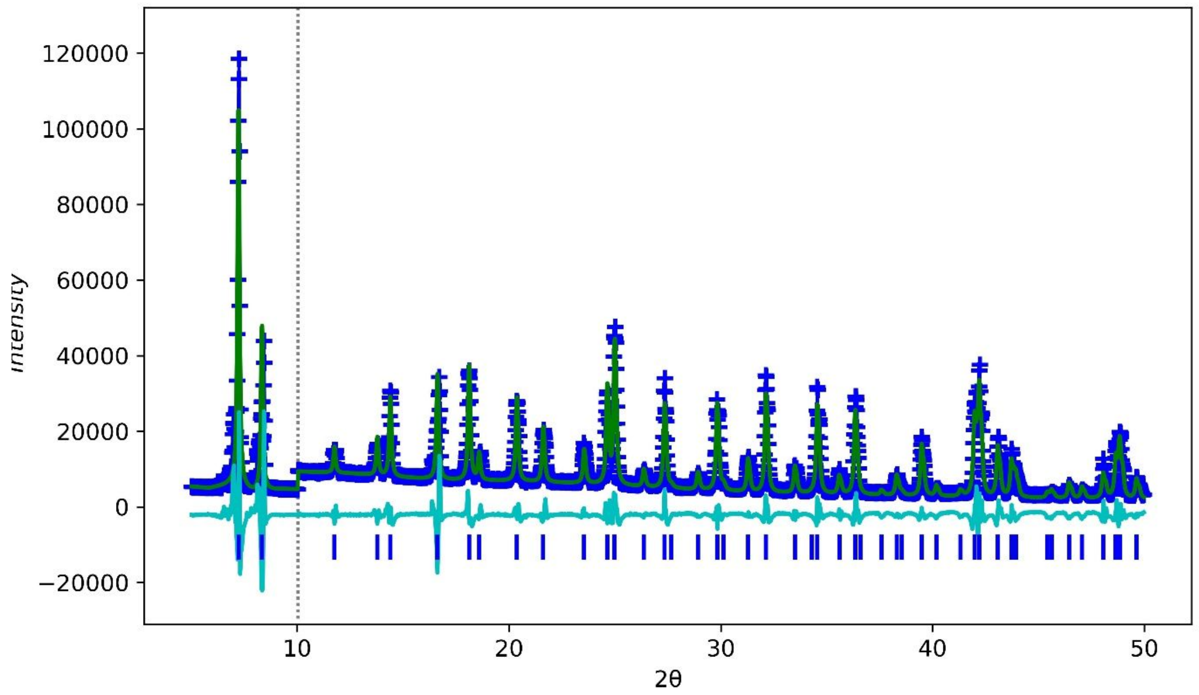

Figure S1. The final observed (blue crosses), calculated (green line) and difference plot (cyan line) for the LeBail refinement of **1·H₂O** at 298 K (cubic *Fm*$\bar{3}$*m*, *a* = 21.4398(6) Å, *V* =9855.2(8) Å^3, R_{wp} = 18.54 %, R_p = 11.11 %). Reflection markers are shown in blue and the higher angle portion of the pattern is magnified 2 fold.

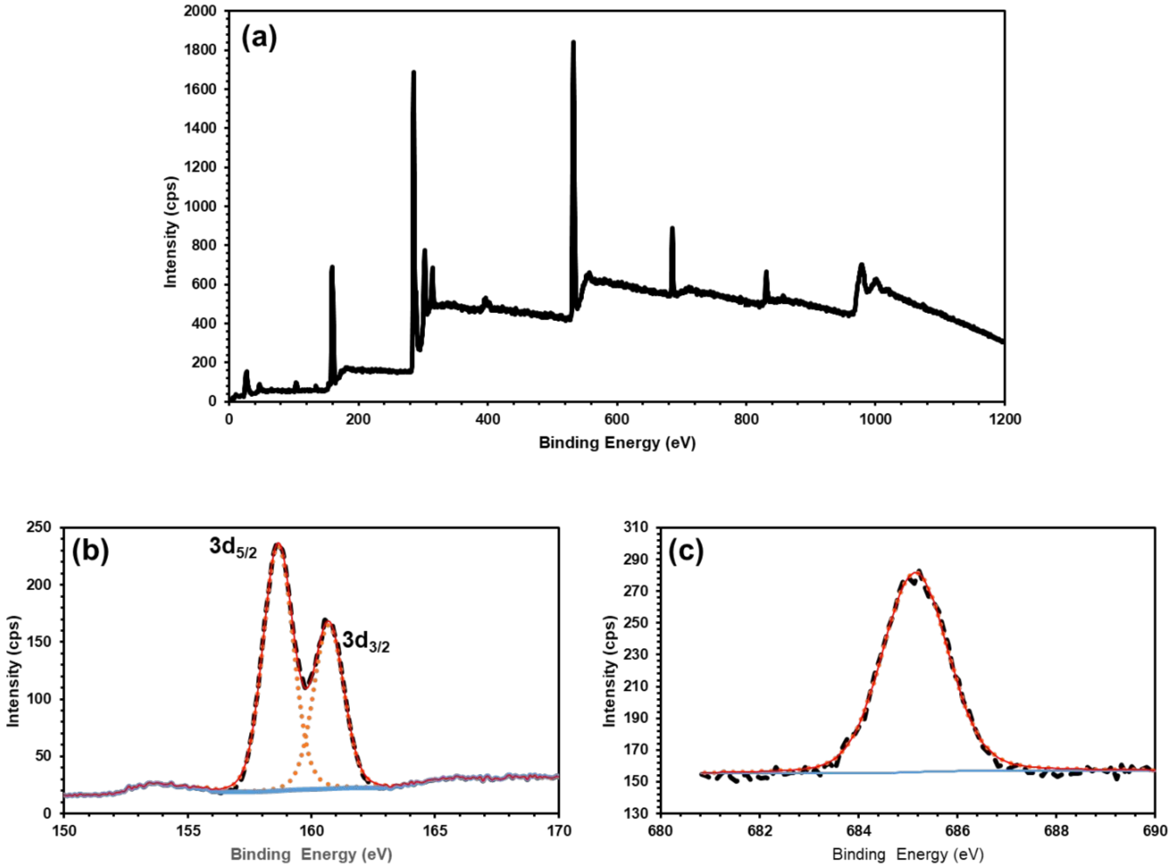

Figure S2. XPS survey (a), Y3d (b) and F1s (c) spectra for 1·H_2O. Key: Observed data- black dashes or line, contributed fitted peaks – orange circles, total envelope fit – red and background-blue.

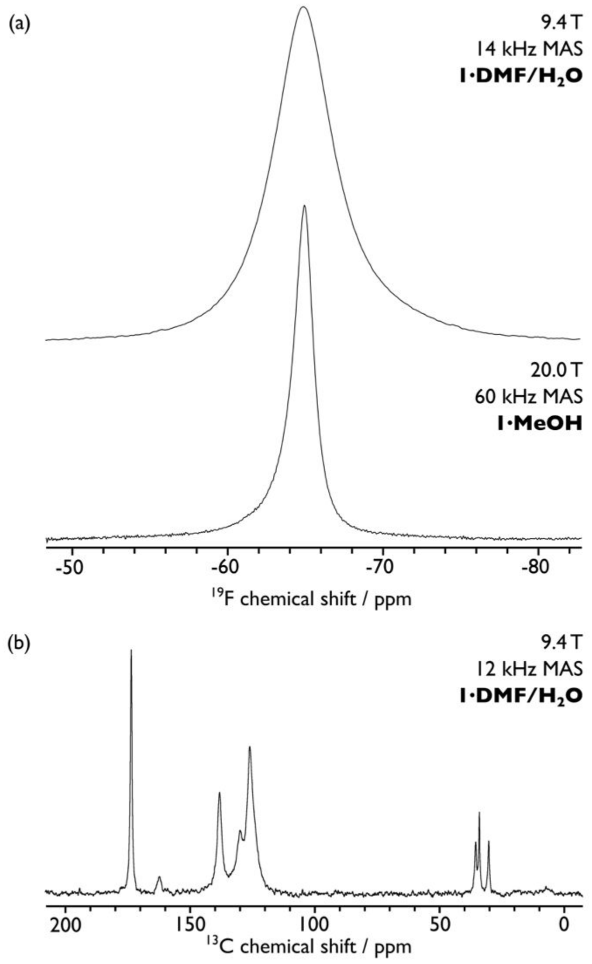

Figure S3. ^{19}F MAS NMR spectra of 1·DMF/H_2O (top) and 1·MeOH (bottom) recorded at 9.4 and 20.0 T using MAS frequencies of 14 and 60 kHz, respectively, showing a single fluorine environment at $\delta\{^{19}F\}$ = –65 ppm (a). {^{1}H-}^{13}C CPMAS NMR spectrum of 1·DMF/H_2O, recorded at 9.4 T using an MAS frequency of 12 kHz, showing ^{13}C resonances from the ndc linker as well as from DMF ($\delta\{^{13}C\}$ = 163, 36, and 31 ppm) and DMA ($\delta\{^{13}C\}$ = 34 ppm) (b).

Table S3. Comparison of atomic displacement parameters and final residual values for 1·DMF/H_2O with 100 % O(H) occupancy and 100 % F occupancy of atoms on the μ_3-X bridging position at 100 K

Element on μ_3-X bridging position (see Figure 2a for reference)	U_{eq} μ_3-X1 (Å^2)	U_{eq} μ_3-X2 (Å^2)	R_1 (%)	wR_2 (%)
O	0.0015(6)	0.0014(6)	6.8	24.5
F	0.0111(6)	0.0111(7)	6.2	21.9

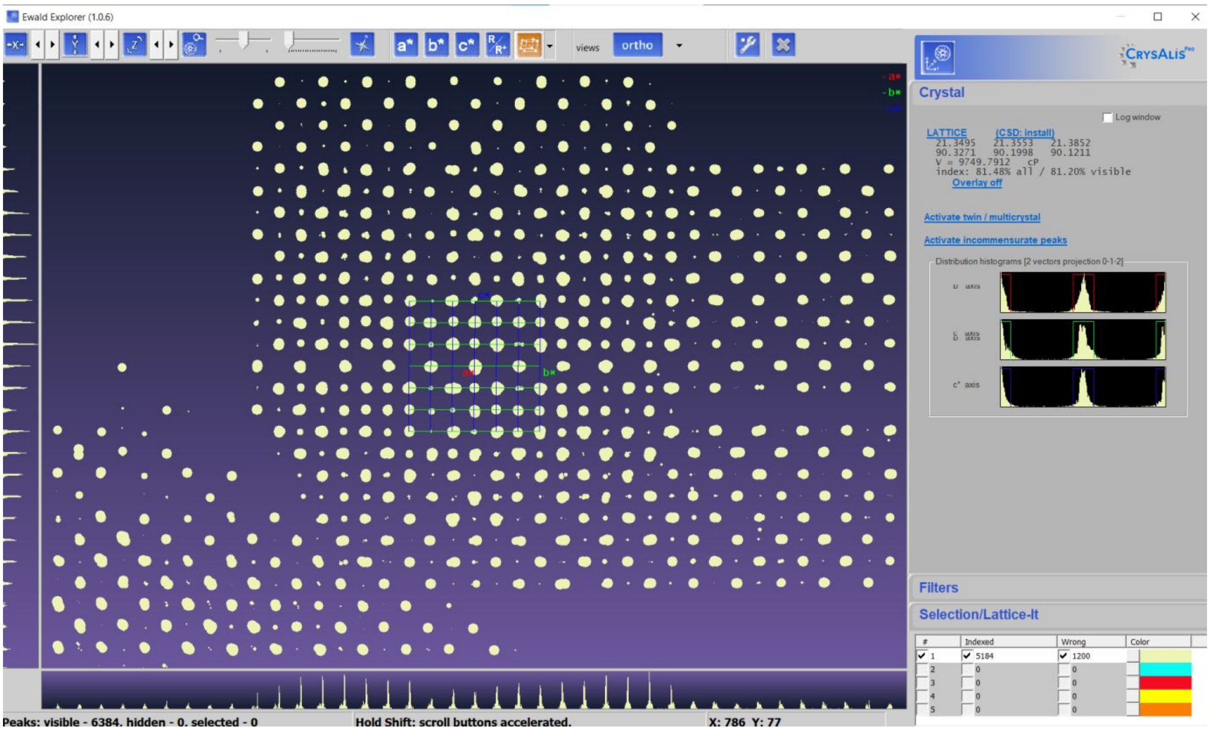

Figure S4a: Ewald sphere projection along a* for **1·DMF/H_2O**.

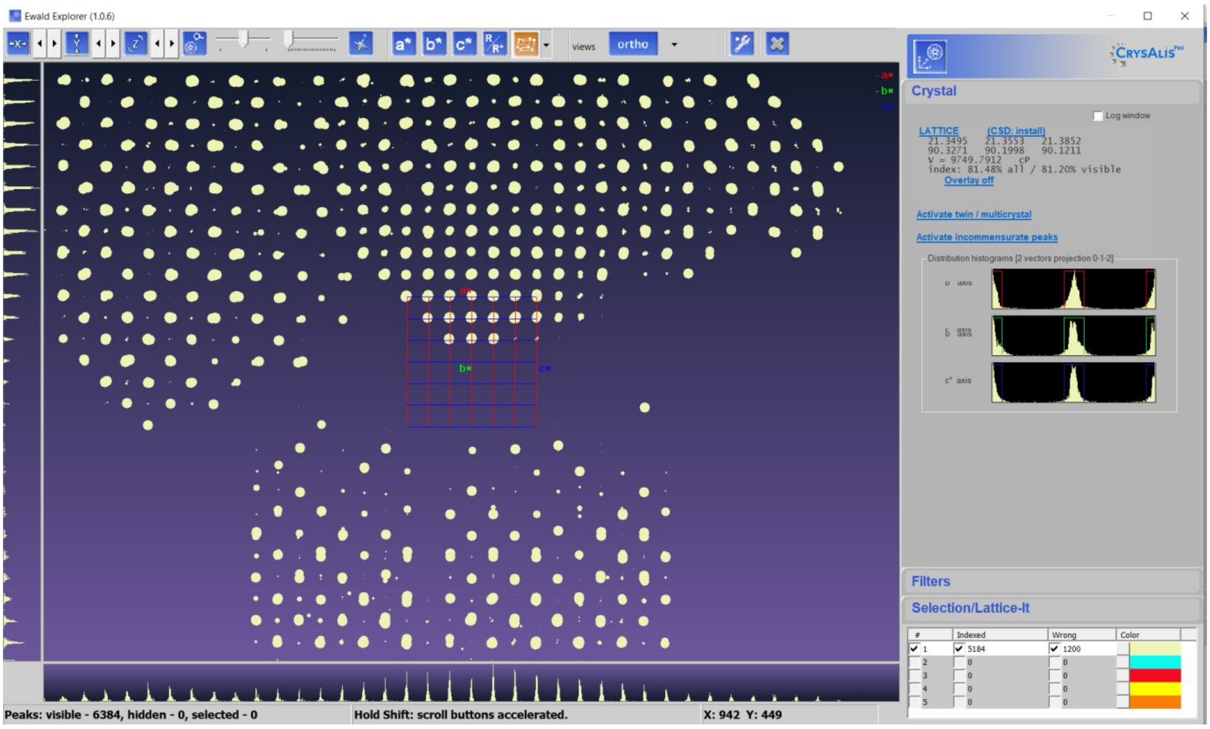

Figure S4b: Ewald sphere projection along b* for **1·DMF/H_2O**.

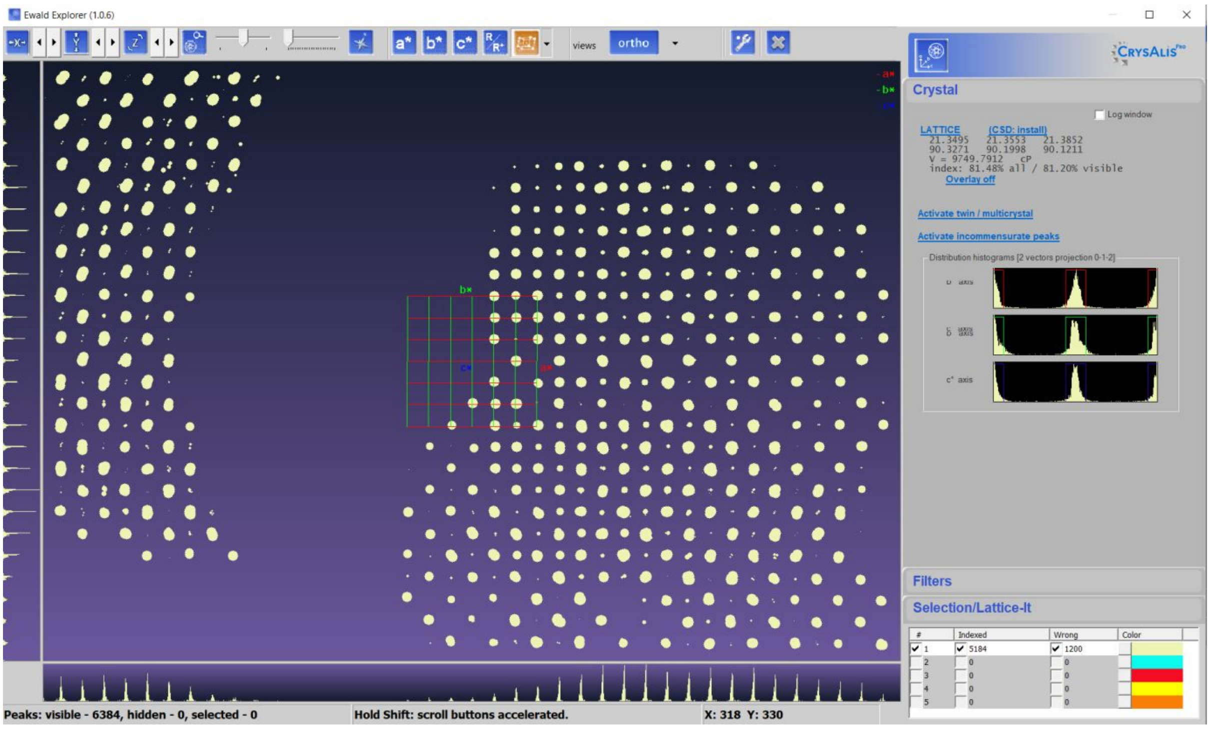

Figure S4c: Ewald sphere projection along c* for **1·DMF/H_2O**.

Table S4: Number of reflections breaking the possible lattice centering reflection conditions for 1·DMF/H_2O

	P	A	B	C	I	F	Obverse	Reverse	All
**Number (total)**	0	6804	6806	6822	6750	10216	8992	9015	13517
**Number I>3σ**	0	3383	3668	3505	4334	5278	5633	5660	8508
**<I>**	0	1.2	1.2	1.1	36.9	1.2	36.9	37	36.9
**<I/σ>**	0	7.6	8	7.5	26.9	7.7	25.4	25.5	25.6

Table S5: Number of reflections breaking the possible glide planes and screw axes reflection conditions for 1·DMF/H_2O

	42=21	41/43	a--	b--	n--	--n
**Number (total)**	16	24	640	640	618	814
**Number I>3σ**	0	8	6	180	182	628
**<I>**	0	355.8	0	1	1	136
**<I/σ>**	0.3	37.2	0.1	6.5	6.8	550.6

Table S6: Comparison of R_{int} merging statistics for 1·DMF/H_2O reduced as a single component crystal in space groups $Pa\overline{3}$ and $Fm\overline{3}m$.

Space group	R_{int} (%)
$Pa\overline{3}$ (205)	1.43
$Fm\overline{3}m$ (225)	1.60

Table S7: Number of reflections breaking the possible lattice centering reflection conditions for 1·MeOH (data provided as a comparator for a crystal structure of 1 with space group $Fm\overline{3}m$)

	P	A	B	C	I	F	Obverse	Reverse	All
Number (total)	0	6436	6455	6455	6450	9673	8541	8566	12807
Number I>3σ	0	9	5	4	1591	9	2081	2108	3129
<I>	0	0	0	0	43.8	0	44	44.5	44
<I/σ>	0	0.1	0.1	0.1	15.4	0.1	15	15.1	15

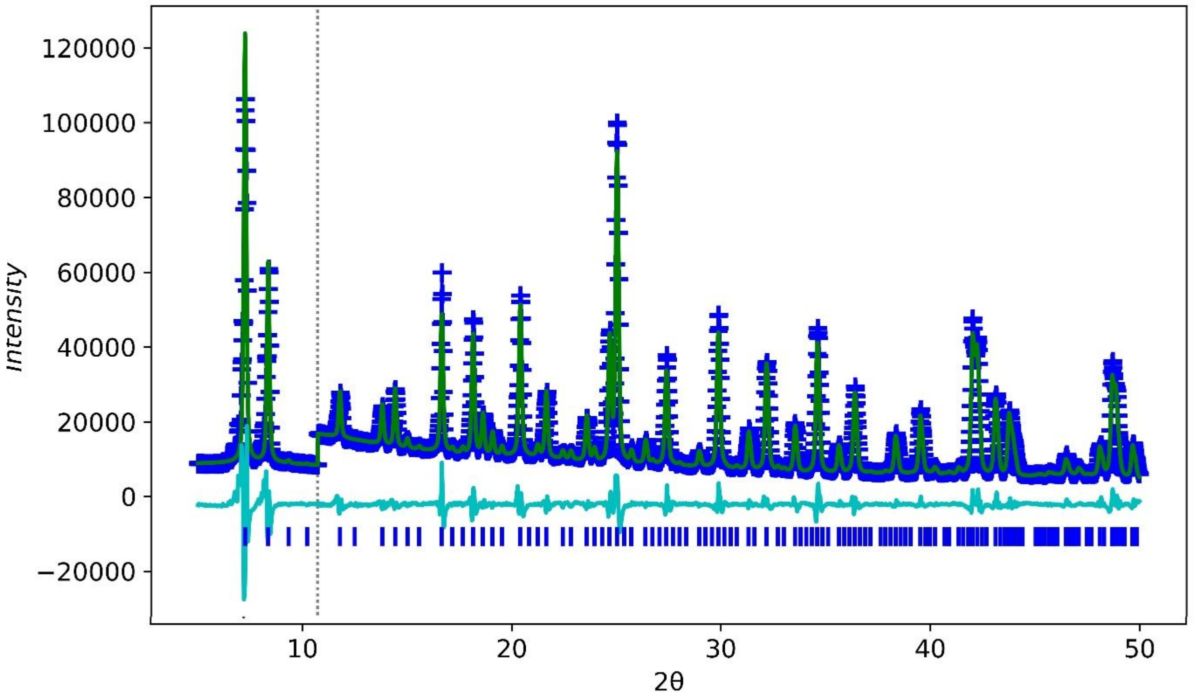

Figure S5. The final observed (blue crosses), calculated (green line) and difference plot (cyan line) for the LeBail refinement of **1·DMF/H_2O** at 298 K (cubic *Pa*$\bar{3}$, *a* = 21.441(1) Å, *V* =9857(1) Å^3, R_{wp} = 10.62 %, R_p = 6.22 %). Reflection markers are shown in blue and the higher angle portion of the pattern is magnified 2 fold.

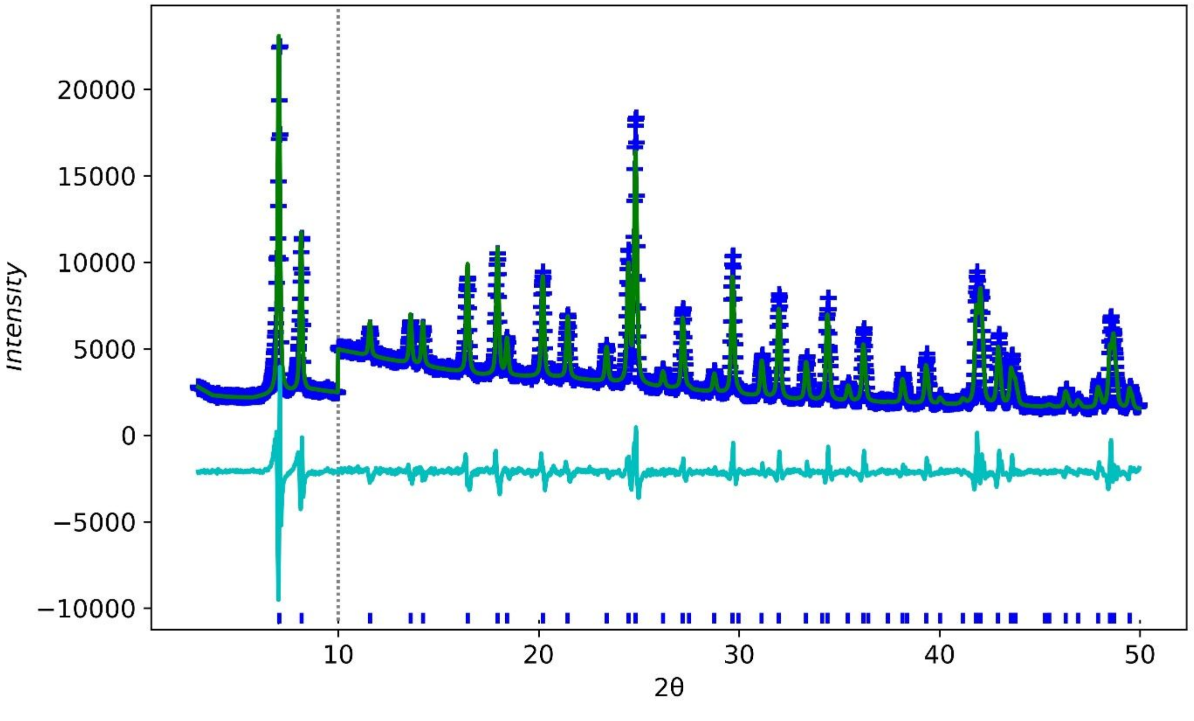

Figure S6. The final observed (blue crosses), calculated (green line) and difference plot (cyan line) for the LeBail refinement of **1·MeOH** at 298 K (cubic *Fm*$\bar{3}$*m*, *a* = 21.4381(5) Å, *V* =9852.7(7) Å^3, R_{wp} = 11.51 %, R_p = 7.22 %). Reflection markers are shown in blue and the higher angle portion of the pattern is magnified 2 fold.

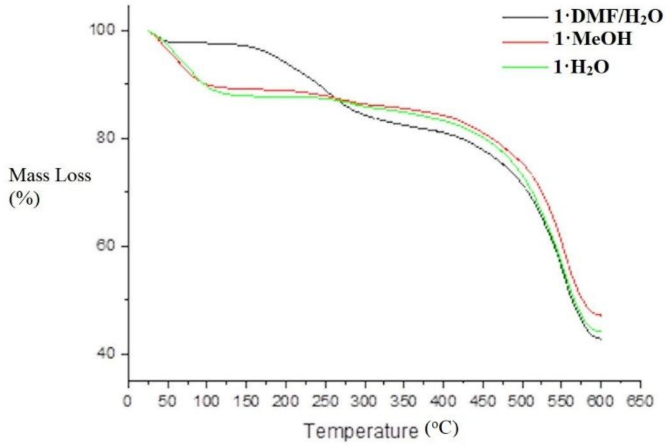

Figure S7. TGA traces **1·DMF/H_2O**, **1·MeOH** and **1·H_2O**.

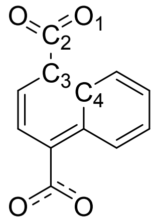

Figure S8. A diagram of the ndc linker showing the atoms, $O_1C_2C_3C_4$, used to define the torsion angle **j (°)** listed in Table 2.
